# A machine learning framework for predicting cognitive impairment in aging populations using urinary metal and demographic data

**DOI:** 10.3389/fgene.2025.1631228

**Published:** 2025-06-25

**Authors:** Fengchun Ren, Xiao Zhao, Qin Yang, Huaqiang Liao, Yudong Zhang, Xuemei Liu

**Affiliations:** ^1^ Department of Radiology, Hospital of Chengdu University of Traditional Chinese Medicine, Chengdu, Sichuan, China; ^2^ Department of Radiology, Chongqing Hospital of Jiangsu Province Hospital, The People’s Hospital of Qijiang District, Chongqing, China; ^3^ Department of Infectious Diseases, Affiliated Hospital of North Sichuan Medical College, Nanchong, Sichuan, China

**Keywords:** machine learning, cognitive impairment, metal, demographic, SHAP

## Abstract

**Introduction:**

Cognitive impairment in older adults poses a significant global public health concern, with environmental metal exposure emerging as a major risk factor. However, the combined effects of multiple metals and the modulatory roles of demographic variables remain insufficiently explored.

**Methods:**

This study analyzed data from four NHANES cycles (1999–2000, 2001–2002, 2011–2012, 2013–2014), comprising 1,230 participants aged ≥ 60 years. Urinary concentrations of nine metals and creatinine were quantified in conjunction with demographic variables. Cognitive status was classified using data-driven quartile thresholds on the Digit Symbol Substitution Test, CERAD Word-Learning Test, and Animal Fluency tests. Six machine learning algorithms were trained and evaluated using sensitivity (SN), specificity (SP), accuracy (ACC), Matthews correlation coefficient (MCC) and AUC.

**Results:**

The eXtreme gradient boosting (XGBoost) model demonstrated superior performance across all metrics (SN = 0.78, SP = 0.84, ACC = 0.81, MCC = 0.62, AUC = 0.90), and was selected for subsequent interpretation. SHAP analysis identified educational level, age, race/ethnicity, and creatinine as primary predictors. Elevated thallium and molybdenum levels and reduced barium levels also contributed to cognitive risk. Ultimately, a user-friendly webserver was deployed for the predictive model and is freely accessed at http://bio-medical.online/admxp/.

**Discussion:**

The associated webserver enables accessible risk screening and underpins precision prevention strategies in aging populations.

## 1 Introduction

Cognitive impairment in older adults constitutes a significant and escalating global public health challenge, with profound implications for quality of life and healthcare provision (Javitt, 2023; Petersen et al., 2018). A growing body of evidence implicates environmental metal exposure as a contributor to cognitive decline. For example, Gao et al. demonstrated that aluminum exposure induces cognitive deficits by promoting abnormal tau phosphorylation via activation of the ERK signaling pathway (Gao et al., 2025). Similarly, Li et al. reported that elevated urinary levels of barium, cadmium, lead, and tungsten were associated with diminished cognitive scores, whereas higher levels of molybdenum, cobalt, strontium, and thallium correlated positively with cognitive performance among older adults in the United States (Li et al., 2025). These findings highlight the complex, metal-specific effects of environmental exposure on cognitive health. However, most existing studies have focused on the individual metals, thereby overlooking potential synergistic or antagonistic interactions among multiple metals that may jointly influence cognitive function (Wang et al., 2022; Qing et al., 2024; Li et al., 2023).

Urinary metal concentrations have emerged as valuable biomarkers for assessing both acute and chronic metal exposure, owing to their noninvasive collection and ability to reflect total body burden (Qing et al., 2024; Price, 1982; Martinez-Morata et al., 2023). Shafie and Ashour provided a comprehensive review of advanced colorimetric and fluorometric techniques for urinary cadmium detection, thereby underscoring the relevance of urinary biomarkers for monitoring metal toxicity in vulnerable populations, particularly children (Shafie and Ashour, 2025). In a subsequent study, Domingo-Relloso et al. found that elevated urinary levels of arsenic, cobalt, copper, uranium, and zinc correlated with associated with poorer performance on cognitive tests and an elevated risk of dementia among older adults (Domingo-Relloso et al., 2024).

Large-scale population-based investigations have further substantiated the link between environmental metal exposure and adverse cognitive outcomes. Wu et al. analyzing NHANES 2011–2014 data, reported that urinary cadmium was negatively associated with cognitive performance, whereas selenium exerted a protective effect (Wu et al., 2024). Similarly, Liu et al. undertook a cross-sectional study in China and found that elevated plasma levels of iron and zinc were positively associated with cognitive function, whereas higher nickel and lead levels were inversely associated with cognitive performance (Hong et al., 2024).

Despite recent advances, the interactive effects of multiple urinary metals, as well as the modulatory roles of demographic factors (e.g., age, sex, education), remain inadequately explored. Moreover, predictive models that integrate such multidimensional variables to identify individuals at risk of cognitive dysfunction are limited. To address these gaps, this study aimed to construct a predictive model for cognitive impairment in older adults by integrating urinary metal profiles with demographic characteristics derived from NHANES data. A suite of machine learning algorithms and SHAP (SHapley Additive exPlanations) interpretation techniques were employed to improve model transparency and predictive accuracy. Additionally, an accessible online analysis platform was developed to enable users to input urinary metal concentrations and demographic variables for estimating the risk of cognitive dysfunction. This platform facilitates routine cognitive health monitoring and may serve as an early screening tool for high-risk populations, offering advantages over conventional neuropsychological assessments in terms of convenience and scalability. Ultimately, the study advances precision prevention of cognitive decline and informs targeted intervention strategies for aging populations.

## 2 Materials and methods

### 2.1 Sample collection

**FIGURE 1:**
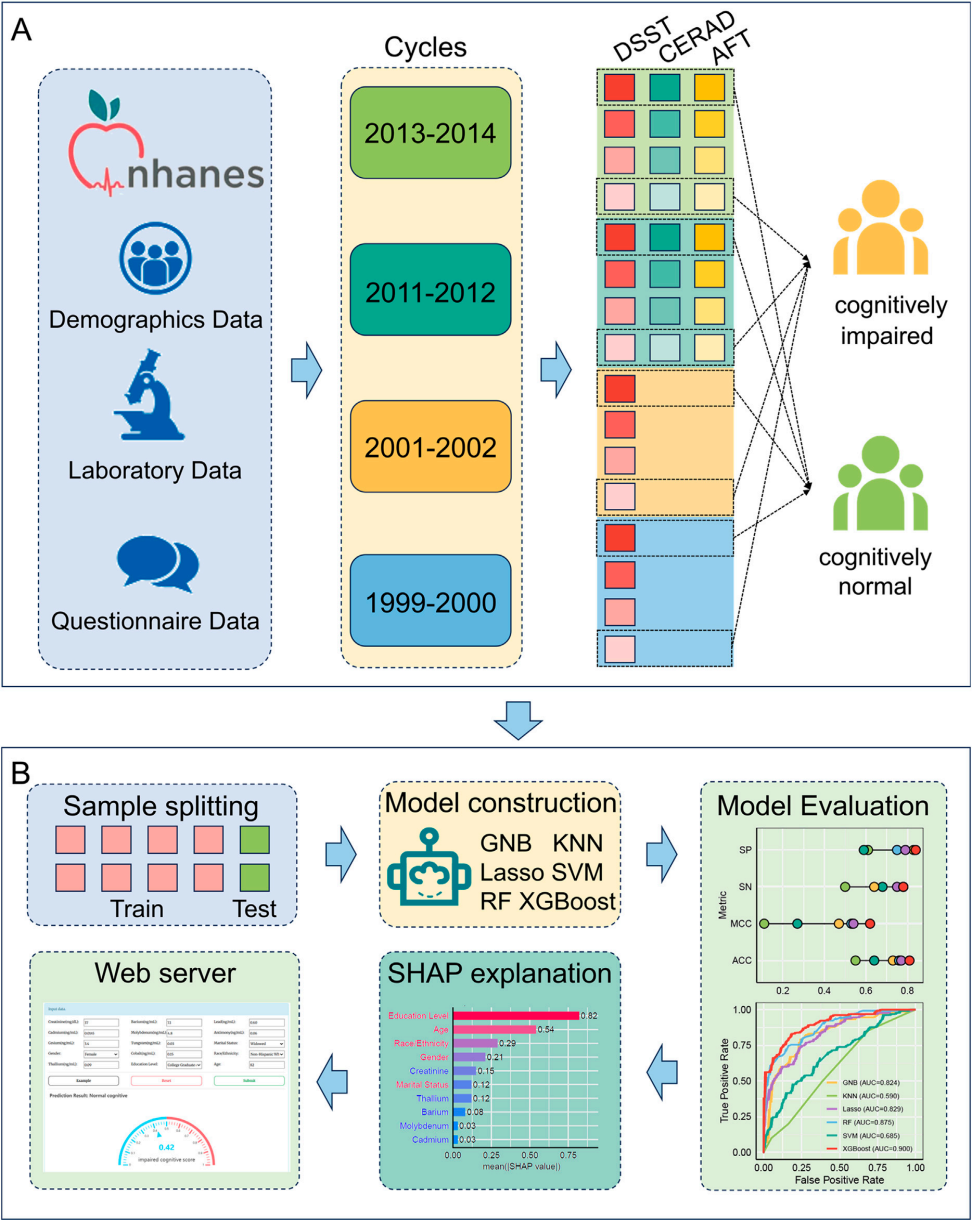
The workflow for the study. **(A)** data collection and processing flow, **(B)** predictive modeling process.

We obtained demographic, questionnaire, and laboratory data from four NHANES cycles (1999–2000, 2001–2002, 2011–2012, and 2013–2014), merged records via the unique participant identifier, and restricted our sample to participants aged 60 years or older with at least one completed cognitive assessment. In the 1999–2000 and 2001–2002 cycles, only Digit Symbol Substitution Test (DSST) scores were available, whereas the 2011–2012 and 2013–2014 cycles additionally incorporated the CERAD Word‐Learning Test and Animal Fluency Test (AFT). Each participant’s laboratory profile comprised ten urinary markers, including creatinine (mg/dL) and nine metals quantified in ng/mL (Barium, Cadmium, Cobalt, Cesium, Molybdenum, Lead, Antimony, Thallium, and Tungsten). This panel provides a comprehensive assessment of metal exposure. These procedures yielded four harmonized analytic cohorts for subsequent predictive modeling (Figure 1).

We classified cognitive function using data‐driven quartile thresholds, given the absence of universally accepted cut-offs for CERAD, AFT, or DSST scores. Previous NHANES-based studies similarly defined cognitive impairment as scores within the lowest quartile for each test, and multiple investigations have utilized the 25th percentile of DSST raw scores to identify low performers (Wang et al., 2022; Gong et al., 2021). Accordingly, in each of the four age-eligible (≥ 60 years) NHANES cohorts (1999–2000, 2001–2002, 2011–2012, 2013–2014), participants scoring below the 25th percentile on any administered test were classified as “cognitively impaired”, whereas those above the 75th percentile were classified as having normal cognitive function. This classification approach yielded 651 cognitively impaired and 579 cognitively normal individuals (total N = 1,230), which were carried forward as our positive and negative sets. Then the dataset was randomly partitioned into training and testing sets using an 8:2 ratio for the development and evaluation of machine learning models.

### 2.2 Measurement of urinary metals levels

Urinary metal concentrations were measured using inductively coupled plasma mass spectrometry (ICP-MS), a highly sensitive analytical technique capable of detecting trace elements at concentrations as low as parts per trillion. Urine samples were prepared by diluting them in a 1:9 ratio with 2% (v/v) double-distilled, concentrated nitric acid containing iridium and rhodium as internal standards. These internal standards help correct for potential signal drift and matrix effects during analysis, ensuring accurate quantification of metal concentrations. The prepared samples were then introduced into the ICP-MS instrument, where the high-temperature argon plasma ionizes the sample, and the resulting ions are separated and detected based on their mass-to-charge ratios. This method allows for precise and simultaneous quantification of multiple metals in biological samples, making it particularly suitable for biomonitoring studies involving trace metal exposure.

### 2.3 Machine learning algorithms

Machine learning techniques have become integral to biomarker discovery and disease diagnosis, offering advanced capabilities for analysing complex biological data (Zhou et al., 2025; Yang et al., 2024; Kang et al., 2020; Liu XW. et al., 2024; Yang Z. et al., 2023; Nayarisseri et al., 2021; Levin et al., 2017; Liu et al., 2024b; Sun et al., 2022; Pan et al., 2024). In this study, six machine learning algorithms, including Gaussian Naive Bayes (GNB), Random Forest (RF), K Nearest Neighbors (KNN), Support Vector Machine (SVM), Lasso and eXtreme Gradient Boosting (XGBoost) were employed to estimate the risk of cognitive dysfunction. All models were trained on the training dataset and their performance evaluated on the hold-out testing dataset. During model training, five-fold cross-validation was employed, and hyperparameter optimization was performed using the GridSearchCV function from the scikit-learn library. The optimal hyperparameter configurations and their corresponding search ranges for each model are summarized in Supplementary Table S1.

### 2.4 Evaluation of machine learning model

Model performance was assessed using multiple evaluation metrics (Pham et al., 2023a; Pham et al., 2023b; Zou et al., 2023), including sensitivity (SN), specificity (SP), accuracy (ACC), and Matthew’s correlation coefficient (MCC), as defined in Equation 1.

$$\left\{\begin{array}{l} SN=\frac{TP}{TP+FN}\\ SP=\frac{TN}{TN+FP}\\ ACC=\frac{TP+TN}{TP+TN+FN+FP}\\ MCC=\frac{TP*TN-FP*FN}{\sqrt{\left(TN+FP\right)\left(TN+FN\right)\left(TP+FP\right)\left(TP+FN\right)}} \end{array}\right.$$ (1)

where TP, TN, FP, and FN denote the numbers of true positives, true negatives, false positives, and false negatives, respectively. Moreover, the area under the receiver operator curve (AUC) and receiver operating characteristic curve (ROC) analysis were used to access model performance (Zulfiqar et al., 2023; Chen et al., 2024).

### 2.5 Interpretable methods of prediction model based on SHAP

SHAP analysis provides an explanation framework for individual predictions (Lundberg and Lee, 2017; Yang H. et al., 2023; Wang et al., 2023). It originates from the Shapley value concept in cooperative game theory. The objective is to elucidate a given instance prediction by quantifying each feature contribution. In this study, SHAP is primarily employed to rank model variables by importance within the developed framework. SHAP can effectively interpret each variable contribution to the model and mitigate the enduring “black box” issue in machine learning.

### 2.6 Statistical analysis

In the baseline analysis, categorical variables (“Gender,” “Race/Ethnicity,” “Education Level,” and “Marital Status”) were compared between cognitive impairment and normal cognition groups using Pearson’s chi-square test, while continuous variables were assessed via the Wilcoxon rank-sum test. For these continuous measures, group-specific means and standard deviations were calculated. All statistical analyses were conducted using R (version 4.4.1), ensuring consistent implementation of chi-square and nonparametric testing procedures commonly employed in NHANES-based studies.

## 3 Results

### 3.1 Baseline characteristics

We analysed data from 1,230 participants (579 cognitively normal and 651 cognitively impaired) and observed that key demographic factors. As shown in Table 1, sex, race/ethnicity, education level, marital status, and age were all significantly associated with cognitive status across cohorts. Higher educational level demonstrated the strongest association (p = 4.45E-64), reflecting large‐scale evidence that extended years of schooling confer resilience against cognitive decline. Among urinary biomarkers, creatinine, barium, cadmium, lead, antimony, and thallium exhibited significant between-group differences. These observations align with prior NHANES‐based analyses linking elevated urinary heavy metals to impaired performance on speeded cognitive tests (Wang et al., 2022). These results underscore that integrating demographic variables and urinary metal profiles markedly improves the predictive accuracy of models designed to identify older adults at high risk for cognitive impairment.

**TABLE 1:** Characteristics of the study population.

Characteristics	Overall (n = 1,230)	Cognitive normal (n = 579)	Cognitive impairment (n = 651)	P value
Gender				7.61E-06
Male	609	247	362	
Female	621	332	289	
Race/Ethnicity				1.75E-23
Mexican American	159	43	116	
Other Hispanic	101	27	74	
Non-Hispanic White	587	364	223	
Non-Hispanic Black	279	96	183	
Other Race	104	49	55	
Education Level				4.45E-64
Less Than 9th Grade	238	14	224	
9-11th Grade	173	51	122	
High School Grad/GED	273	135	138	
Some College or AA degree	284	179	105	
College Graduate or above	258	199	59	
Refused	1	1	0	
Don’t Know	3	0	3	
Marital Status				3.67E-06
Married	704	376	328	
Widowed	259	88	171	
Divorced	147	68	79	
Separated	29	8	21	
Never married	68	27	41	
Living with partner	21	11	10	
Refused	2	1	1	
Age	69.84 (7.10)	67.72 (6.20)	71.73 (7.32)	5.21E-22
Creatinine (mg/dL)	104.77 (67.61)	97.98 (63.50)	110.81 (70.56)	9.30E-04
Barium (ng/mL)	1.63 (3.10)	1.61 (2.00)	1.65 (3.82)	2.55E-04
Cadmium (ng/mL)	0.52 (0.67)	0.45 (0.44)	0.59 (0.81)	7.59E-04
Cobalt (ng/mL)	0.53 (1.63)	0.53 (1.80)	0.53 (1.46)	1.56E-01
Cesium (ng/mL)	4.87 (3.23)	4.99 (3.43)	4.76 (3.03)	4.32E-01
Molybdenum (ng/mL)	50.79 (47.12)	50.98 (51.56)	50.62 (42.83)	8.95E-02
Lead (ng/mL)	0.87 (1.26)	0.72 (0.73)	1.01 (1.58)	2.20E-02
Antimony (ng/mL)	0.09 (0.19)	0.09 (0.23)	0.09 (0.14)	2.56E-02
Thallium (ng/mL)	0.16 (0.14)	0.17 (0.12)	0.16 (0.14)	1.89E-02
Tungsten (ng/mL)	0.11 (0.27)	0.11 (0.31)	0.11 (0.22)	3.70E-01
AFT Score	16.49 (6.14)	20.85 (4.92)	12.74 (4.34)	3.08E-88
CERAD Score	24.44 (7.50)	29.72 (4.27)	20.00 (6.70)	5.97E-83
DSST score	44.90 (20.55)	61.37 (12.46)	29.34 (13.26)	1.35E-159

### 3.2 Evaluation and comparison of model

**FIGURE 2:**
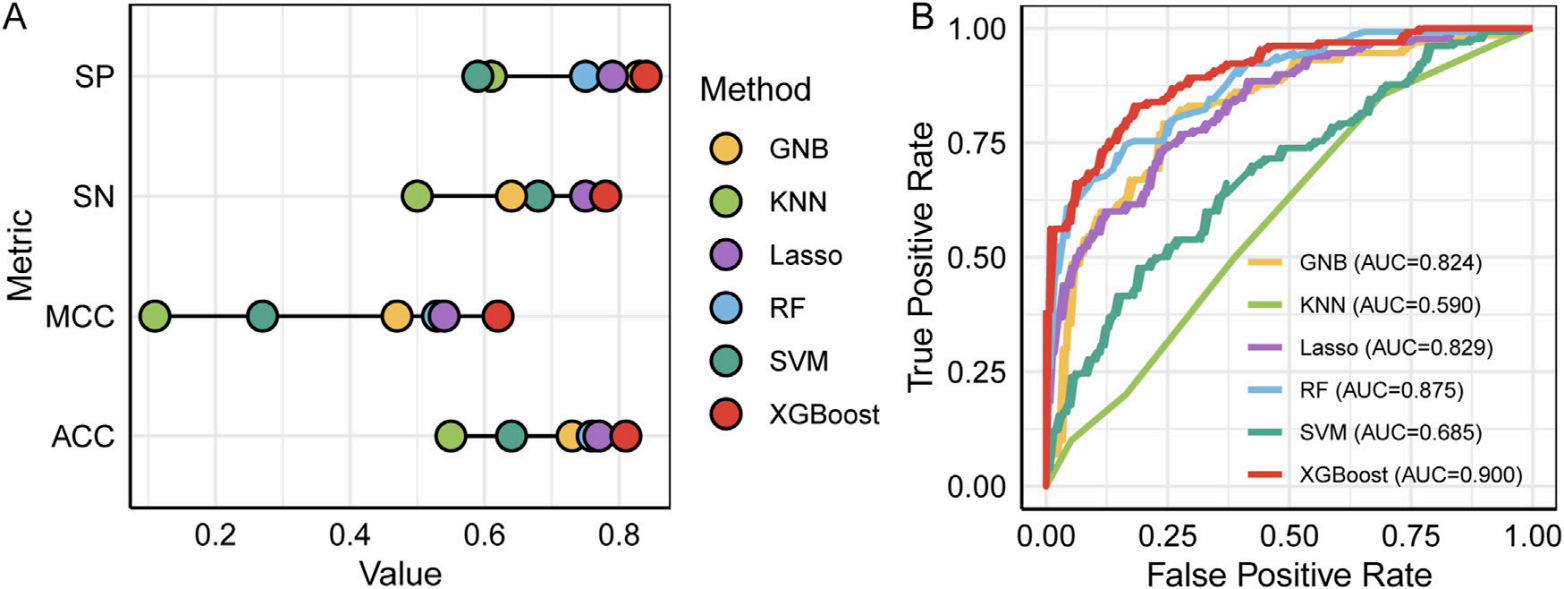
Performance comparison of different prediction models. **(A)** SP, SN, MCC and ACC, **(B)** the ROC and AUC.

All models were trained on an identical training dataset and evaluated on a common testing dataset using the features sets summarized in Table 1. As shown in Figure 2A and detailed Table 2, the XGBoost model outperformed all other algorithms across all evaluation metrics. Specifically, XGBoost achieved a SN of 0.78, representing a 28% improvement over KNN, and a SP of 0.84, representing a 23% increase relative to KNN. Compared with the RF model, which ranked second in ACC and MCC, XGBoost improved ACC by 5% and MCC by 9%. The ROC and AUC values are presented in Figure 2B, where the XGBoost model demonstrates the best predictive performance, achieving an AUC of 0.90, at least 2.5% higher than the other models. Therefore, the predictive model based on XGBoost was ultimately selected for subsequent analyses.

**TABLE 2:** Performance comparison of six models on the testing dataset.

Method	SN	SP	ACC	MCC
GNB	0.64	0.83	0.73	0.47
KNN	0.50	0.61	0.55	0.11
RF	0.78	0.75	0.76	0.53
SVM	0.68	0.59	0.64	0.27
Lasso	0.75	0.79	0.77	0.54
XGBoost	0.78	0.84	0.81	0.62

### 3.3 Visualization of feature importance

**FIGURE 3:**
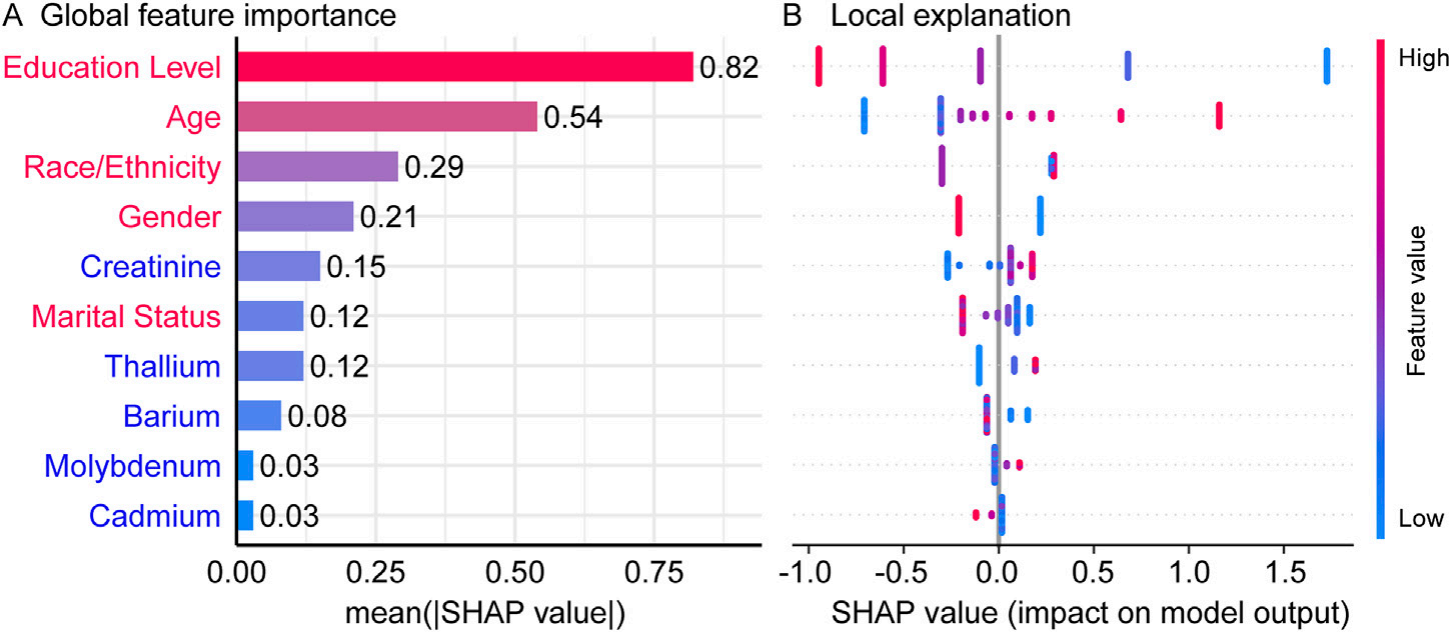
The SHAP summary plot. **(A)** ranking of feature importance, with demographic features in red and urinary markers in blue, **(B)** the SHAP summary plot of all variables and cognitive impairment risk.

Following the initial analysis, SHAP was applied to interpret the XGBoost-based predictive model. Permutation feature importance analysis provided insights into the relative significance of all variables within the model (Liu et al., 2023). As shown in Figure 3A, the top ten contributing variables were identified, with Education level, Age, Race/Ethnicity, Gender and Creatinine emerging as the most influential features for predicting impaired cognitive function. Furthermore, the associations between the top ten variables and predicted cognitive impairment risk were validated. The SHAP summary plot (Figure 3B) illustrates the overall effect of heavy metals and baseline variables on cognitive impairment risk, ranked in descending order of feature importance. In this context, a positive SHAP value indicates a positive association between feature value and impaired cognitive risk, with larger values corresponding to greater contribution. Among demographic characteristics, educational level exhibited an inverse association with cognitive impairment risk, whereas age demonstrated a positive association. Additionally, the cognitive impairment risk was higher in men than in women. Overall, demographic factors emerge as the primary influences on cognitive dysfunction.

The SHAP analysis identified a significant positive correlation between urinary creatinine levels and the risk of cognitive impairment. This finding aligns with previous research indicating that elevated urinary albumin-to-creatinine ratio (UACR) is associated with an increased risk of cognitive decline, even at low UACR levels (Teng et al., 2025; Liu et al., 2024c; Ahmed et al., 2024). Renal dysfunction, as indicated by abnormal creatinine levels, may contribute to cognitive impairment through various mechanisms, such as the accumulation of neurotoxic substances due to impaired kidney function, leading to neuronal damage and cognitive decline.

Additionally, the SHAP analysis revealed a positive association between high urinary levels of thallium and molybdenum and the risk of cognitive impairment. This observation is consistent with prior studies demonstrating that exposure to multiple heavy metals, including thallium and molybdenum, is linked to decreased cognitive performance (Fu et al., 2024). These metals may exert neurotoxic effects through mechanisms such as oxidative stress, inflammation, and direct neuronal toxicity, ultimately leading to cognitive deficits (Zhang et al., 2024). Conversely, we found that low urinary barium level was associated with an increased risk of cognitive impairment. This finding is supported by research indicating that lower barium concentrations, in conjunction with higher exposure to other metals like lead and cadmium, may exacerbate neurotoxic effects and contribute to cognitive decline (Li et al., 2025). These results suggest that barium may have a protective or antagonistic role in the context of multi-metal exposure, and its deficiency could enhance the toxicity of other metals, negatively impacting cognitive function.

### 3.4 Webserver implementation

**FIGURE 4:**
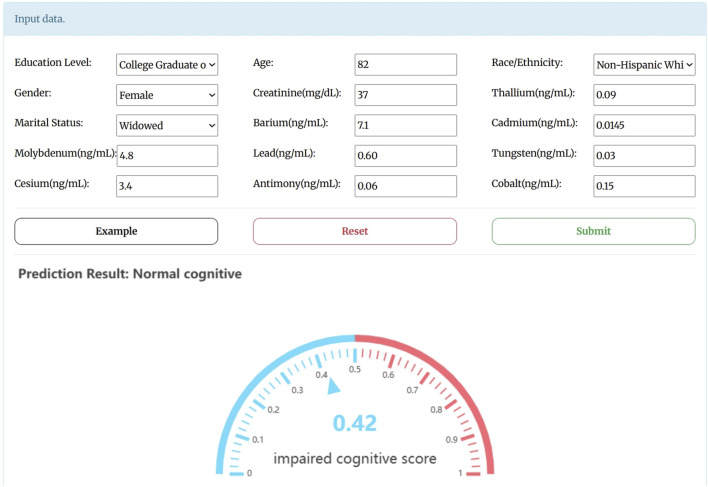
The homepage for the webserver.

To facilitate researchers access, a user-friendly webserver was deployed for our predictive model, freely accessed at http://bio-medical.online/admxp/. The server’s homepage is presented in Figure 4. The step-by-step instructions for using the webserver are as follows: users can input the relevant characterization indicators into the input box or use the sample input provided by the “Example” button. They can then click the “Submit” button to obtain prediction results. A resulting score greater than 0.5 indicates cognitive dysfunction, while a score of 0.5 or below indicates normal cognitive function.

## 4 Discussion

In this study, we demonstrated that integrating urinary metal biomarkers with demographic characteristics markedly improves the prediction of cognitive impairment in older adults. By applying XGBoost on harmonized NHANES data and interpreting the model with SHAP and permutation feature importance, we identified educational level, age, race/ethnicity, and Gender as the foremost determinants of cognitive decline risk, with elevated levels of cadmium, lead, thallium, and other heavy metals exerting additional contributory effects. The superior performance of the XGBoost model (AUC = 0.90, SN = 0.78, SP = 0.84) underscores its utility for early, non-invasive screening, outperforming traditional neuropsychological tests in scalability and objectivity.

Moreover, the deployment of a user-friendly web server provides an accessible platform for researchers and clinicians to estimate individual risk profiles in real time, facilitating routine cognitive health monitoring and guiding precision prevention strategies. Our findings highlight the critical role of socio-environmental factors in geriatric cognitive health and advocate for multifactorial risk assessment frameworks. Future work should validate this model in longitudinal cohorts, explore additional environmental exposures and genetic moderators, and assess the impact of targeted interventions—such as educational programs or heavy-metal chelation—on attenuating cognitive decline. Collectively, this integrative approach lays the groundwork for personalized screening and intervention paradigms to mitigate the growing burden of age-related cognitive disorders.

Despite the promising findings, several limitations should be acknowledged. First, the cognitive assessments used in this study were limited to individuals aged 60 years and older, in accordance with the NHANES protocol. Consequently, the predictive model developed herein is applicable only to this demographic, thereby limiting its generalizability to younger populations.

Second, although the integration of demographic characteristics with urinary metal biomarkers offers a robust predictive framework, the relatively limited sample size and feature set restricted the use of deep learning methodologies. Deep learning models generally require large-scale datasets to achieve optimal performance. The current sample size may be inadequate for training complex deep neural networks without incurring the risk of overfitting or reduced generalizability.

Third, the exclusive reliance on NHANES data for both model training and evaluation constitutes a limitation. Although cross-validation techniques were employed to mitigate overfitting, the lack of external validation on independent cohorts limits the ability to assess the model’s generalizability across diverse populations and settings.

Fourth, the use of NHANES data across multiple survey cycles introduces potential cohort effects and temporal variability. Despite efforts to harmonize variable definitions and apply consistent preprocessing procedures, unmeasured temporal shifts in participant characteristics, exposure sources, or laboratory methodologies may have influenced the results. For example, temporal changes in public health policies, industrial emissions, or dietary habits could affect urinary metal concentrations. Furthermore, periodic revisions to NHANES laboratory protocols or cognitive assessment procedures may introduce inter-cycle variability. Although integrating data from multiple cycles enhances generalizability, temporal dynamics remain a potential source of bias.

In summary, despite the aforementioned limitations, this study provides valuable insights into the early prediction of cognitive impairment in older adults and establishes a foundation for future research.

## Data Availability

Publicly available datasets were analyzed in this study. This data can be found here: https://wwwn.cdc.gov/nchs/nhanes/Default.aspx.

## References

[B1] Ahmed Z. Shahzadi K. Jin Y. Li R. Momanyi B. M. Zulfiqar H. (2024). Identification of RNA-Dependent liquid-liquid phase separation proteins using an artificial intelligence strategy. Proteomics 24 (21-22), e2400044. 10.1002/pmic.202400044 38824664

[B2] Chen X. Zhou S. Yang L. Zhong Q. Liu H. Zhang Y. (2024). Risk prediction of diabetes progression using big data mining with multifarious physical examination indicators. Diabetes Metab. Syndr. Obes. 17, 1249–1265. 10.2147/DMSO.S449955 38496004 PMC10942017

[B3] Domingo-Relloso A. McGraw K. E. Heckbert S. R. Luchsinger J. A. Schilling K. Glabonjat R. A. (2024). Urinary metal levels, cognitive test performance, and dementia in the multi-ethnic study of atherosclerosis. JAMA Netw. Open 7 (12), e2448286. 10.1001/jamanetworkopen.2024.48286 39621345 PMC11612832

[B4] Fu Z. Xu X. Cao L. Xiang Q. Gao Q. Duan H. (2024). Single and joint exposure of Pb, Cd, Hg, Se, Cu, and Zn were associated with cognitive function of older adults. Sci. Rep. 14 (1), 28567. 10.1038/s41598-024-79720-5 39558028 PMC11574263

[B5] Gao D. Yin J. Zhang Y. Li C. Zhao L. Wang L. (2025). The molecular mechanism of miRNA-195-5p regulating ERK involvement in abnormal phosphorylation of tau protein by aluminum maltol in PC12 cells. J. Appl. Toxicol. 10.1002/jat.4795 40273949

[B6] Gong Z. Song W. Gu M. Zhou X. Tian C. (2021). Association between serum iron concentrations and cognitive impairment in older adults aged 60 years and older: a dose-response analysis of national health and nutrition examination survey. PLoS One 16 (8), e0255595. 10.1371/journal.pone.0255595 34339453 PMC8328322

[B7] Hong S. Wu S. Wan Z. Wang C. Guan X. Fu M. (2024). Associations between multiple metals exposure and cognitive function in the middle-aged and older adults from China: a cross-sectional study. Environ. Res. 263 (Pt 1), 120038. 10.1016/j.envres.2024.120038 39305974

[B8] Javitt D. C. (2023). Cognitive impairment associated with schizophrenia: from pathophysiology to treatment. Annu. Rev. Pharmacol. Toxicol. 63, 119–141. 10.1146/annurev-pharmtox-051921-093250 36151052

[B9] Kang J. Yu S. Lu S. Xu G. Zhu J. Yan N. (2020). Use of a 6-miRNA panel to distinguish lymphoma from reactive lymphoid hyperplasia. Signal Transduct. Target Ther. 5 (1), 2. 10.1038/s41392-019-0097-y 32296019 PMC6946694

[B10] Levin N. M. B. Pintro V. O. de Avila M. B. de Mattos B. B. De Azevedo W. F. Jr (2017). Understanding the structural basis for inhibition of cyclin-dependent kinases. New pieces in the molecular puzzle. Curr. Drug Targets 18 (9), 1104–1111. 10.2174/1389450118666161116130155 27848884

[B11] Li J. He X. Gao S. Liang Y. Qi Z. Xi Q. (2023). The metal-binding protein atlas (MbPA): an integrated database for curating metalloproteins in all aspects. J. Mol. Biol. 435 (14), 168117. 10.1016/j.jmb.2023.168117 37086947

[B12] Li Z. Lin Y. Wang W. Xie M. Jiang Y. Wang Z. (2025). Association between mixture exposure to metals in urine and cognitive function in older adults in the United States: NHANES 2011-2014. J. Trace Elem. Med. Biol. 89, 127643. 10.1016/j.jtemb.2025.127643 40245653

[B13] Liu M. Zhou J. Xi Q. Liang Y. Li H. Liang P. (2023). A computational framework of routine test data for the cost-effective chronic disease prediction. Brief. Bioinform 24 (2), bbad054. 10.1093/bib/bbad054 36772998

[B14] Liu T. Huang J. Luo D. Ren L. Ning L. Huang J. (2024b). Cm-siRPred: predicting chemically modified siRNA efficiency based on multi-view learning strategy. Int. J. Biol. Macromol. 264 (Pt 2), 130638. 10.1016/j.ijbiomac.2024.130638 38460652

[B15] Liu T. Qiao H. Wang Z. Yang X. Pan X. Yang Y. (2024c). CodLncScape provides a self-enriching framework for the systematic collection and exploration of coding LncRNAs. Adv. Sci. (Weinh) 11 (22), e2400009. 10.1002/advs.202400009 38602457 PMC11165466

[B16] Liu X. W. Li H. L. Ma C. Y. Shi T. Y. Wang T. Y. Yan D. (2024a). Predicting the role of the human gut microbiome in type 1 diabetes using machine-learning methods. Brief. Funct. Genomics 23 (4), 464–474. 10.1093/bfgp/elae004 38376798

[B17] Lundberg S. M. Lee S.-I. (2017). A unified approach to interpreting model predictions. Proc. 31st Int. Conf. Neural Inf. Process. Syst., 4768–4777. 10.5555/3295222.3295230

[B18] Martinez-Morata I. Sobel M. Tellez-Plaza M. Navas-Acien A. Howe C. G. Sanchez T. R. (2023). A state-of-the-science review on metal biomarkers. Curr. Environ. Health Rep. 10 (3), 215–249. 10.1007/s40572-023-00402-x 37337116 PMC10822714

[B19] Nayarisseri A. Khandelwal R. Tanwar P. Madhavi M. Sharma D. Thakur G. (2021). Artificial intelligence, big data and machine learning approaches in precision medicine and drug discovery. Curr. Drug Targets 22 (6), 631–655. 10.2174/1389450122999210104205732 33397265

[B20] Pan X. Ren L. Yang Y. Xu Y. Ning L. Zhang Y. (2024). MCSdb, a database of proteins residing in membrane contact sites. Sci. Data 11 (1), 281. 10.1038/s41597-024-03104-7 38459036 PMC10923927

[B21] Petersen R. C. Lopez O. Armstrong M. J. Getchius T. S. D. Ganguli M. Gloss D. (2018). Practice guideline update summary: mild cognitive impairment [RETIRED]: report of the guideline development, dissemination, and implementation subcommittee of the American academy of neurology. Neurology 90 (3), 126–135. 10.1212/WNL.0000000000004826 29282327 PMC5772157

[B22] Pham N. T. Phan L. T. Seo J. Kim Y. Song M. Lee S. (2023a). Advancing the accuracy of SARS-CoV-2 phosphorylation site detection *via* meta-learning approach. Brief. Bioinform 25 (1), bbad433. 10.1093/bib/bbad433 38058187 PMC10753650

[B23] Pham N. T. Rakkiyapan R. Park J. Malik A. Manavalan B. (2023b). H2Opred: a robust and efficient hybrid deep learning model for predicting 2'-O-methylation sites in human RNA. Brief. Bioinform 25 (1), bbad476. 10.1093/bib/bbad476 38180830 PMC10768780

[B24] Price R. G. (1982). Urinary enzymes, nephrotoxicity and renal disease. Toxicology 23 (2-3), 99–134. 10.1016/0300-483x(82)90092-0 6126019

[B25] Qing Y. Zheng J. Luo Y. Li S. Liu X. Yang S. (2024). The impact of metals on cognitive impairment in the elderly and the mediating role of oxidative stress: a cross-sectional study in shanghai, China. Ecotoxicol. Environ. Saf. 286, 117152. 10.1016/j.ecoenv.2024.117152 39383823

[B26] Shafie A. Ashour A. A. (2025). Cadmium toxicity in children and its detection using colorimetric and fluorimetric methods. J. Fluoresc. 10.1007/s10895-025-04275-1 40192914

[B27] Sun Y. Li H. Zheng L. Li J. Hong Y. Liang P. (2022). iProbiotics: a machine learning platform for rapid identification of probiotic properties from whole-genome primary sequences. Brief. Bioinform 23 (1), bbab477. 10.1093/bib/bbab477 34849572

[B28] Teng Y. Zhang J. Yang B. Luo Q. Xue Y. Zhang M. (2025). Elevated urine albumin-to-creatinine ratio as a risk factor for cognitive impairment in older adults: a cross-sectional analysis of NHANES data. PLoS One 20 (5), e0321519. 10.1371/journal.pone.0321519 40323962 PMC12052149

[B29] Wang H. Zhang Z. Li H. Li J. Li H. Liu M. (2023). A cost-effective machine learning-based method for preeclampsia risk assessment and driver genes discovery. Cell Biosci. 13 (1), 41. 10.1186/s13578-023-00991-y 36849879 PMC9972636

[B30] Wang X. Xiao P. Wang R. Luo C. Zhang Z. Yu S. (2022). Relationships between urinary metals concentrations and cognitive performance among U.S. older people in NHANES 2011-2014. Front. Public Health 10, 985127. 10.3389/fpubh.2022.985127 36148349 PMC9485476

[B31] Wu L. Xin Y. Zhang J. Yang X. Chen T. Niu P. (2024). Associations between metals, serum folate, and cognitive function in the elderly: mixture and mediation analyses. Environ. Health (Wash). 2 (12), 865–874. 10.1021/envhealth.4c00071 39722838 PMC11667285

[B32] Yang H. Luo Y. M. Ma C. Y. Zhang T. Y. Zhou T. Ren X. L. (2023b). A gender specific risk assessment of coronary heart disease based on physical examination data. NPJ Digit. Med. 6 (1), 136. 10.1038/s41746-023-00887-8 37524859 PMC10390496

[B33] Yang X. Wu C. Liu W. Fu K. Tian Y. Wei X. (2024). A clinical-information-free method for early diagnosis of lung cancer from the patients with pulmonary nodules based on backpropagation neural network model. Comput. Struct. Biotechnol. J. 24, 404–411. 10.1016/j.csbj.2024.05.010 38813092 PMC11134880

[B34] Yang Z. Cai X. Ye Q. Zhao Y. Li X. Zhang S. (2023a). High-throughput screening for the potential inhibitors of SARS-CoV-2 with essential dynamic behavior. Curr. Drug Targets 24 (6), 532–545. 10.2174/1389450124666230306141725 36876836

[B35] Zhang Y. Yang Y. Ren L. Zhan M. Sun T. Zou Q. (2024). Predicting intercellular communication based on metabolite-related ligand-receptor interactions with MRCLinkdb. BMC Biol. 22 (1), 152. 10.1186/s12915-024-01950-w 38978014 PMC11232326

[B36] Zhou Y. Liu W. Luo C. Huang Z. Samarappuli Mudiyanselage Savini G. Zhao L. (2025). Ab-Amy 2.0: predicting light chain amyloidogenic risk of therapeutic antibodies based on antibody language model. Methods 233, 11–18. 10.1016/j.ymeth.2024.11.005 39550021

[B37] Zou X. Ren L. Cai P. Zhang Y. Ding H. Deng K. (2023). Accurately identifying hemagglutinin using sequence information and machine learning methods. Front. Med. (Lausanne) 10, 1281880. 10.3389/fmed.2023.1281880 38020152 PMC10644030

[B38] Zulfiqar H. Guo Z. Ahmad R. M. Ahmed Z. Cai P. Chen X. (2023). Deep-STP: a deep learning-based approach to predict snake toxin proteins by using word embeddings. Front. Med. (Lausanne) 10, 1291352. 10.3389/fmed.2023.1291352 38298505 PMC10829051

