# Supplementary material for: A machine learning framework for predicting cognitive impairment in aging populations using urinary metal and demographic data
Source: Front Genet. 2025 Jun 25;16:1631228. doi: 10.3389/fgene.2025.1631228 (PMC12237647; doi:10.3389/fgene.2025.1631228)
Supplement: Supplementary file 1 [file Table1.docx]

Table S1. Hyperparameter optimization range of the models.

| Model | Parameters |
| --- | --- |
| KNN | n_neighbors: range from 1 to 10 with step 1 |
| GNB | Default |
| Lasso | alpha: [0.1, 0.2, 0.3, 0.4, 0.5, 0.6, 0.7, 0.8, 0.9] |
| SVM | C: [0.001, 0.01, 0.1, 1, 10, 100, 1000],  gamma: [ 100, 10, 1, 0.1, 0.01, 0.001, 0.0001] |
| RF | criterion: ['gini', 'entropy'],  n_estimators: range from 10 to 100 by step 1,  max_depth: range from 1 to 10 by step 1,  min_samples_split: range from 2 to 25 by step 1,  min_samples_leaf: range from 1 to 10 by step 1,  max_leaf_nodes: range from 1 to 120 by step 1,  ccp_alpha: 20 spaced evenly on a log scale using the numpy.logspace with start=10, stop=0 and base=10 |
| XGBOOST | n_estimators: range from 10 to 100 by step 1,  max_depth: range from 1 to 10 by step 1,  min_child_weight: range from 1 to 10 by step 1,  gamma: range from 0.01 to 1 by step 0.01,  subsample: range from 0 to 1 by step 0.1,  colsample_bytree: range from 0 to 1 by step 0.1,  reg_lambda: range from 0 to 100 by step 1,  reg_alpha: range from 0 to 10 by step 1,  eta: 40 spaced evenly on a log scale using the numpy.logspace with start=-2, stop=0 and base=10 |
